# An Ideal Elementary School

**Published:** 1920-09-25

**Authors:** 


### An Ideal Elementary School.

Some time ago we tried to picture in these columns an ideal school play-centre of the future. Now Mr. E. A. Craddock, in *The Teachers' World*, describes an actual school at a typical industrial town which he calls "Marwich" that as nearly approaches the ideal as any educational institution of the kind that one could expect to find in this drab world of modern industry. The headmaster, who has "the brow of a visionary and the mouth of a man of deeds," starts out with this explicit aim: "We try," he says, "to teach the value and grace of quiet movement, the virtues of water and the sweetness of neat and ordered habit. We have brought these children into contact with men and women of high ideals and depth of culture, that they may feel the fire of enthusiasm and see radiating from a living presence the charm of knowledge."

Fine words, perhaps, but note how the parsnips are buttered. The school has been placed outside the town boundaries, and such value does an unusually enlightened education authority place upon education that it has arranged to "subsidise the excellent tramway service for the daily conveyance to and from school of children over seven years of age." The school is in a country village, where each class, in a separate building, creates its own individuality. The central building is reached from these "class houses" by means of fruit-laden gardens and flowery paths. The dinners are provided in a communal dining-room by the labour of the scholars, and here brightness, cleanliness and good manners are intended "to render these boys and girls dissatisfied with the primitive customs and surroundings which characterise most of their homes."

In addition to creating such an atmosphere of charm, this extraordinary headmaster has actually induced the authority to raise the scale of salaries for elementary school teachers to the level of that paid to secondary staffs in the borough, with the result that men and women of the highest education are ready to help in the new school and to co-operate with the head in his imaginative outlook. Mr. Craddock's comment on the experiment is as follows: "If these children do not in a few years scrape away the smudgy houses of 'Marwich' and let light and colour into its drab streets, I shall be very much surprised."

There are two practical questions we should like to ask on this experiment: (1) Where precisely is "Marwich?" and (2) How much does it cost the local authority to administer the school? If "Marwich" is really typical, and if the cost is not really out of proportion to the power of the community to bear it, a case will have been made out for a personal visit in the spirit of honest inquiry from the President of the Board of Education himself and for a series of deputations from the authorities of all industrial towns to learn how to produce a healthy race in the next generation.

